# The challenge of using intermittent preventive therapy with sulfadoxine/pyrimethamine among pregnant women in Uganda

**DOI:** 10.1186/s12936-016-1462-8

**Published:** 2016-08-09

**Authors:** Humphrey Wanzira, Henry Katamba, Allen Eva Okullo, Denis Rubahika

**Affiliations:** 1 National Malaria Control Programme, Ministry of Health, Kampala, Uganda; 2 School of Public Health, Fellowship Program, Makerere University, Kampala, Uganda

**Keywords:** Intermittent preventive therapy in pregnancy, Sulfadoxine/pyrimethamine, Malaria in pregnancy

## Abstract

**Background:**

The Uganda National Malaria Control Programme recommends the use of intermittent preventive therapy in pregnancy with sulfadoxine/pyrimethamine (SP) to prevent malaria, however, there is overwhelming evidence of low uptake of this intervention. This study, therefore, sought to examine the factors associated with taking two or more doses of therapy among women who had had the most recent live birth.

**Methods:**

This was a secondary data analysis of the 2014 Malaria Indicator Survey dataset. The outcome was the use of two or more doses of SP for the most recent live birth while independent variables included; age, highest education attained, residence (rural and urban), use of radio and community health teams for malaria related messages, knowledge of taking SP and use of LLINS to prevent malaria, household wealth, skilled attendant seen at ANC and number of children the woman has.

**Results:**

Of the 1820 women included in the final analysis, 822 (45.16 %) women took two or more doses of SP. Women who knew that this therapy was used to prevent malaria and those who had been seen by a skilled attendant were 10.72 times [Adjusted OR (95 % CI): 10.72 (7.62–15.08), p-value = 0.001] and 3.19 times [Adjusted OR (95 % CI): 3.19 (1.26–8.07), p-value  = 0.015] more likely to take at least two doses as compared to those who did not know about this therapy and those seen by unskilled attendants, respectively.

**Conclusion:**

This study shows that knowledge among women that SP is a medication used for malaria prevention during pregnancy increases the uptake of two or more doses of this therapy among pregnant women. This highlights the importance of behaviour change communication focused on IPTp uptake that can be complemented by having skilled personnel attending to pregnant women at the antenatal clinic.

## Background

Malaria is hyper-endemic in over 90 % of Uganda’s regions [1] and, therefore, still remains a disease of public health importance. The greatest burden of this disease is born by populations who have either not yet acquired full immunity to malaria, like children under 5 years, or those whose immunity has been suppressed, for example by physiological conditions like pregnancy among women [1, 2].

The National Malaria Control Programme recognizes the significance of increasing the coverage of the most important malaria control interventions and use especially in these two vulnerable populations. In the past decade, with increasing funding, there has been a scale-up of interventions especially on LLIN coverage, case management, and intermittent preventive therapy in pregnant women (IPTp) using sulfadoxine/pyrimethamine (SP). Such efforts have included the shift to universal LLIN coverage [1, 3], with a mass campaign in 2013, increasing availability of rapid diagnostic tools (RDTs) and artemisinin-based combination therapy (ACT) at health facilities for case management and IPTp using SP among pregnant mothers at the ante-natal clinics(ANC) [1]. The national guidelines recommend at least four ANC visits for all pregnant mothers in line with the WHO guidelines [4, 5], where they receive an LLIN on their first visit, and also take SP at each of the visits [6].

So far, there has been success in LLIN coverage and use, with 90 % of households owning at least an LLIN and 75 % of pregnant women sleeping under one the night before the survey. There has also been considerable improvement regarding case management where mothers of 82 % of children with fever, sought treatment or advice and 87 % of children with a fever were treated with an artemisinin-based combination therapy (ACT). However, this success has noticeably been slow with IPTp uptake among pregnant women, with 89 % of women receiving one or more doses of SP, and this reduces to 45 % who received two or more doses and further down to 25 %who received three or more doses [7]. This trend is not only peculiar to Uganda, but has also been reported in other African countries [8, 9].

This is of concern in the fight against malaria among pregnant, a disease whose consequences are clearly documented to include anaemia in mothers, still birth, early neonatal death, pre-term delivery, intra-uterine growth retardation and low birth weight in infants [10–12]. The malaria prevention benefits of taking SP as IPTp to avoid such adverse events are known [13, 14] and, therefore, its scale up is of great importance. To reduce the burden of malaria in pregnancy, a combination of all these interventions should be implemented to protective population coverage levels [5, 6] and the NMCP has adopted this strategy with the aim of achieving 85 % intervention coverage in the populations at risk by 2020 [1].

Therefore, using the Uganda 2014 malaria indicator survey (MIS 2014), the objectives of this secondary analysis was to determine the factors associated with taking two or more doses of SP among women who had had a live pregnancy 2 years prior to the survey. This would give an understanding of individual related factors that lead to the low uptake of the IPTp in this setting thereby contributing to the design and implementation of evidence based strategies to improve the SP usage coverage.

## Methods

This was a secondary data analysis using a dataset from the recently concluded 2014 Uganda Malaria Indicator Survey.

### Description of the malaria indicator Survey (MIS) dataset

The survey was conducted during the months of December 2014 and January 2015 [7]. Households were selected using a stratified two-stage cluster design from 210 enumeration areas, representing all the regions of the country with 44 in urban areas and 166 in rural areas. An EA was defined as a natural village in rural areas and a city block in urban areas. In the first stage, 20 sampling strata (derived from 10 regional domains: Central 1 and 2, East Central, Kampala, Mid-Northern, Mid-Western, Mid-Eastern, South-Western and West Nile) were created and EAs were selected independently from each stratum by a probability-proportional-to-size selection. In the selected EAs, a complete listing of households and a mapping exercise was conducted in November 2014, with the resulting list of households serving as the sampling frame for the selection of households in the second stage. The average EA size was 94 households in urban areas and 77 households in rural areas, with an overall average size of 80 households per EA. In the second stage of the selection process, 28 households were selected in each EA by equal probability systematic sampling. A total of 5802 households were selected for the 2014 MIS, of which 5494 were occupied. Of the occupied households, 5345 were successfully interviewed, yielding a response rate of 97 %. The response rate among households in rural areas was slightly higher (98 %) than the response rate in urban areas (96 %). The main reason for non-response was failure to find individuals at home despite up to four repeated visits to the household. Informed consent was obtained from all participating heads of households and women of child-bearing age in the households that participated in the survey.

For the specifics of this study, a women’s questionnaire administered to all women aged 15–45 years in a selected household was used to collect data on women’s background characteristics (age, education,); reproductive history (number of births, postnatal care etc.); IPTp using SP for malaria during recent pregnancies in the last 2 years; information on knowledge of LLIN use and malaria.

### Study variables

The dependent variable of interest was a woman who had taken two or more doses of SP among those that had had a pregnancy 2 years preceding the survey. Independent variables included; age, highest education attained, residence (rural and urban), use of radio and community health teams for malaria related messages, knowledge of taking SP to prevent malaria, knowledge of use of LLINs to prevent malaria, use of an LLIN, wealth, attendant skill seen at ANC (skilled attendants include: doctors, midwives/nurses and clinical officers) and number of children the woman has.

### Data analysis

Stata version 14 (Statcorp, College Station, Texas, USA) was used for all data analysis. For this study, we used the individual recode DHS dataset of the 2014 MIS. In order to cater for sampling variations, only weighted survey data is presented in this manuscript. This is because of the non-proportional allocation of the sample in the different regional domains at the second sampling stage (28 households were selected in each EA by equal probability systematic sampling), resulting into a sample that was not self-weighting. Weighting factors were calculated based on the population of the selected regional domains and added to the MIS datasets so that any results with the regional weight factored into it would be representative at the national and regional level as well as the survey domain level. Details of how the weighting for the different regional domains was estimated are available in the 2014 MIS report [7]. The distributions of study participant’s baseline characteristics were presented as frequencies with respective proportions. A multivariate logistic regression model with a survey function was used to assess for the factors associated with the use of at least two or more doses of SP to initially derive the crude and then the adjusted odds ratio with the respective confidence interval. In all analyses, a p-value of <0.05 was taken as statistically significant.

## Results

**Fig. 1:**
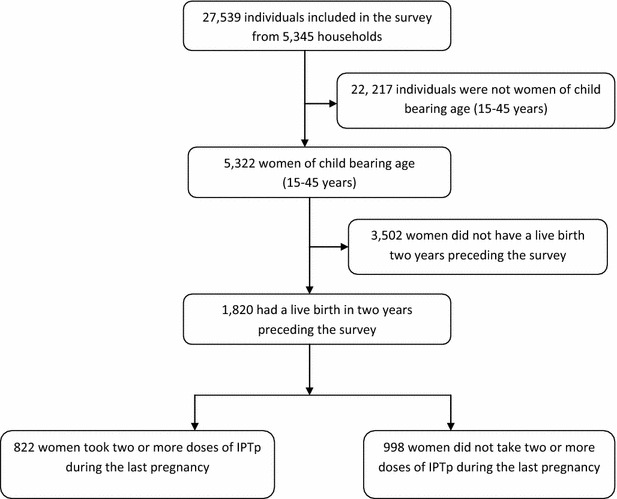
Study profile

Overall, 1820 women, who had had a live birth in the 2 years prior to the survey were included in the final analysis (Fig. 1). As shown in Table 1, the majority of the participants were in the age category of 25–34 years (43.49 %), closely followed by those between 15 and 24 years (41.95 %) and lastly those above 35 years (14.56 %). Women with primary education (60.26 %) and those from rural setting (82.80 %), were the majority for highest education level attained and residence respectively. Overall household wealth and region of origin were more or less with a similar distribution with the exception of those that originated from Kampala that had the least number of women at 4.64 %.

**Table 1:** Women’s baseline characteristics

Characteristic	Distribution of participants	
	Total population N = 1820	Percentage
Age categories (years)		
15–24	763	41.95
25–34	792	43.49
>35	265	14.56
Highest education attained^a^		
No education	294	16.25
Primary	1091	60.26
Secondary	363	20.06
Higher	62	3.43
Residence		
Urban	313	17.20
Rural	1507	82.80
Region		
Central 1	235	12.92
Central 2	174	9.54
East central	215	11.80
Kampala	84	4.64
Mid-North	193	10.61
Mid-Western	212	11.65
Mid-Eastern	189	10.36
North-East	178	9.78
South-Western	194	10.66
West-Nile	146	8.04
Wealth		
Poorest	413	22.69
Poorer	404	22.24
Middle	350	19.22
Richer	323	17.72
Richest	330	18.13

^a^Education missing 9

### The use of two or more doses of SP for IPTp

Of the 1820 women, 1771 (97.31 %) attended at least one ante-natal care visit and of these 97.71 % received SP during their antenatal visit, 1.77 % from another visit to the facility and <1 % (0.53 %) obtained theirs from another source. Overall, 822 of the 1820 (45.16 % [42.88–47.45]) women of child bearing age, who had had a live birth 2 years preceding the survey, took two or more doses of SP.

Among factors considered, only 982 (54.00 %) of women knew that SP was used as a medication to prevent malaria, with the majority, 649 (78.97 %), of these among those who had taken at least two doses of SP. Therefore, the odds of taking two or more doses of SP among women who were knowledgeable regarding SP was 10.72 times greater as compared to those who did not know [Adjusted OR (95 % CI): 10.72 (7.62–15.08), p-value = 0.001] (Table 2). The same was observed among women who had been seen by a skilled attendant at ante-natal clinic, with the odds of taking at least two doses of SP at 3.19 times greater as compared to women who had been seen by an unskilled attendant [Adjusted OR (95 % CI): 3.19 (1.26–8.07), p-value = 0.015]. Similarly women who had three or more children were 1.68 times more likely to take SP [Adjusted OR (95 % CI): 1.68 (1.04–2.70), p-value = 0.033], however, the opposite occurred among women older than 35 years, as they were 38 % less likely to take SP as compared to those between 15 and 24 years [Adjusted OR (95 % CI): 0.62 (0.38–0.99), p-value = 0.043]. The rest of the variables were not statistically significant.

**Table 2:** Factors associated with taking two or more doses of ITPp

Variable	Took two or more doses of IPTp		Crude OR (95 % CI)	Adjusted OR (95 % CI)^a^	p-value
	Yes	No			
Age categories (years)					
15–24	326 (39.68)	437 (43.81)	1	1	
25–34	393 (47.83)	398 (39.91)	1.32 (1.06–1.65)	0.79 (0.53–1.17)	0.233
>35	103 (12.48)	162 (16.28)	0.85 (0.62–1.15)	0.62 (0.38–0.99)	0.043*
Highest education attained					
No education	21 (14.72)	174 (17.52)	1	1	
Primary	449 (54.83)	642 (64.75)	1.01 (0.77–1.32)	0.84 (0.56–1.27)	0.402
Secondary	207 (25.26)	156 (15.76)	1.91 (1.32–2.75)	1.11 (0.62–1.99)	0.728
Higher	43 (5.19)	20 (1.97)	3.14 (1.64–6.00)	1.62 (0.73–3.57)	0.234
Residence					
Urban	158 (19.16)	155 (15.58)			
Rural	664 (80.84)	843 (84.42)	0.78 (0.57–1.06)	1.03 (0.63–1.70)	0.896
Heard malaria messages on radio					
No	100 (18.42)	131 (2.10)			
Yes	441 (81.58)	461 (77.90)	1.26 (0.88–1.79)	1.07 (0.70–1.64)	0.764
Heard malaria messages from CHT					
No	345 (64.20)	361 (61.50)			
Yes	192 (35.80)	226 (38.50)	0.89 (0.64–1.24)	0.86 (0.60–1.23)	0.402
Knowledge of taking IPTp					
No	173 (21.03)	664 (66.59)			
Yes	649 (78.97)	333 (33.41)	7.49 (5.69–9.84)	10.72 (7.62–15.08)	0.001*
Knowledge of use of bed net					
No	155 (19.67)	192 (20.84)			
Yes	635 (80.33)	728 (79.16)	1.08 (0.81–1.42)	1.15 (0.74–1.77)	0.536
Wealth					
Poorest	167 (20.29)	246 (24.67)	1	1	
Poorer	183 (22.27)	222 (22.21)	1.22 (0.91–1.63)	1.16 (0.72–1.87)	0.534
Middle	143 (17.40)	207 (20.71)	1.02 (0.72–1.45)	0.73 (0.45–1.19)	0.211
Richer	148 (18.04)	174 (17.46)	1.26 (0.84–1.88)	1.29 (0.71–2.33)	0.404
Richest	181 (22.00)	149 (14.94)	1.79 (1.23–2.60)	0.96 (0.51–1.81)	0.904
Attendant seen at ANC					
Unskilled attendant	16 (1.94)	67 (6.69)			
Skilled attendant^b^	806 (98.06)	931 (93.31)	3.62 (1.61–8.13)	3.19 (1.26–8.07)	0.015*
Number of hildren					
One	143 (17.35)	212 (21.28)	1	1	0.521
Two	149 (18.08)	174 (17.40)	1.27 (0.91–1.78)	1.19 (0.70–2.04)	0.033*
More than three	531 (64.56)	612 (61.31)	1.29 (1.00–1.66)	1.68 (1.04–2.70)	

*CHT* community health teams, *ANC* antenatal clinic

* Statistically significant at p < 0.05

^a^Adjusted for age, highest education attained, residence (rural and urban), use of radio and community health teams for malaria related messages, knowledge of taking SP was used to prevent malaria, knowledge of use of LLINs to prevent malaria, household wealth, attendant seen at ANC and number of children the woman has

^b^Skilled attendants (doctors, midwives/nurses and clinical officers)

## Discussion

The major finding that women who knew that taking SP to prevent malaria were over ten times more likely to take two or more doses of SP is not surprising considering that the majority, close to 80 %, were knowledgeable regarding the use of SP. This finding is vital especially in this context where just over half the number of the women interviewed knew that SP was a medication used to prevent malaria during pregnancy. This lack of knowledge could possibly be due to a deficiency in behavioural change communication messages that are primarily meant to be delivered at the ante-natal clinic before the medication is prescribed [15, 16]. Of note, this result is in the backdrop that nearly almost all of the women interviewed attended ante-natal clinic with the major if not overall source of SP from the same clinics, an indirect indication that SP is mostly available at these facilities.

In contrast, this study has shown that the knowledge of the use of long-lasting insecticide-treated bed nets (LLIN) among pregnant women is at 80 %, a 35 point increase from the 45 % who took two or more doses of SP, a finding that has already been reported elsewhere [17]. This is despite the fact that both these interventions are administered in the same health facility at the ante-natal clinic in accordance with the national guidelines when on the first ANC visit, mothers are meat to receive an LLIN and also take their first dose of SP, usually at the earliest opportunity of the start of the second trimester [1]. Therefore, one way to improve the knowledge of IPTp as a malaria prevention medication would be to add IPTp malaria prevention messages to the already successful LLINs’ messages, packaged together, especially for pregnant women attending ante-natal clinics. This is an important aspect, considering that for women to be motivated to seek SP for IPTp, it is paramount that they know this therapy is essential for use for malaria prevention, just like LLINs, in pregnancy and it is available free of charge at ante-natal clinics [15, 18].

The finding that more women who have more than three children know the use of SP, possibly due to greater exposure to the messages, and are therefore significantly more likely take at least two doses of SP and also that women who are older than 35 years are less likely to take it, is an indication that these messages could perhaps be emphasized among women with a parity of <3 or those older than 35 years, respectively. Even with this evidence, there is still the possibility that women who took SP were the ones who had experienced its benefits and, therefore, knew of its usefulness in prevention of malaria. This reverse-causality phenomenon is a recognized weakness of cross sectional studies like the MIS, because it is not easy to determine the chronology of events as both outcome and factors associated are measured at the same time.

Another well known finding from this analysis was that women who had been seen by a skilled health worker (medical doctor, clinical officer and nurse/midwife) were close to three times more likely to take at least two doses of SP. This could be because such workers understand the usefulness of appropriate delivery of IPTp malaria prevention messages, in addition to correct prescription, highlighting the importance to improve training of all personnel who attend to pregnant women, as suggested in other studies [19, 20].

The strength of these findings lies in the fact that a large and representative unbiased national sample of women was used, captured in the 2014 MIS. However, there is the weakness that some factors associated with low IPTp uptake were not considered in this analysis since it relied only on individual related variables collected during the survey, leaving out those related to the health system and service providers [21].

## Conclusion

The knowledge that SP is a medication used for malaria prevention during pregnancy is a strong driver of the uptake of at least two or more doses of SP. This could be one of the plausible reasons for the low coverage of this intervention and, therefore, it is important to place emphasis on behaviour change messages directed towards the use of SP in combination with LLINs at ante-natal clinics, coupled with training attending staff on how to deliver such messages in a simple and understandable way. These findings are generalizable to the Ugandan context as the MIS sample size was large and sample from all regions of the country, however, since this analysis was dependant on MIS collected variables, further studies to explore both health system and health provider factors that impact on IPTp usage are recommended.

## Abbreviations

ACTs: artemesinin-based combination therapy
ANC: ante-natal clinic
CHT: Community Health Teams
IPTp: intermittent preventive therapy
LLIN: long lasting insecticide treated bed nets
MIS: Malaria Indicator Survey
NMCP: National Malaria Control Program
SBS-HDREC: Makerere University School of Biomedical Sciences Higher Degrees Research and Ethics Committee
UNCST: Uganda National Council for Science and Technology
VHT: Village Health Team
WHO: World Health Organization
